# Targeting the Checkpoint to Kill Cancer Cells

**DOI:** 10.3390/biom5031912

**Published:** 2015-08-18

**Authors:** Jan Benada, Libor Macurek

**Affiliations:** 1 Department of Cancer Cell Biology, Institute of Molecular Genetics, Academy of Sciences of the Czech Republic, CZ14200 Prague, Czech Republic; E-Mail: jan.benada@img.cas.cz; 2 Department of Genetics and Microbiology, Faculty of Science, Charles University in Prague, CZ12844 Prague, Czech Republic

**Keywords:** checkpoint, DNA damage response, replication stress, cancer, inhibitor, ATM, ATR, Chk1, Wee1, p53

## Abstract

Cancer treatments such as radiotherapy and most of the chemotherapies act by damaging DNA of cancer cells. Upon DNA damage, cells stop proliferation at cell cycle checkpoints, which provides them time for DNA repair. Inhibiting the checkpoint allows entry to mitosis despite the presence of DNA damage and can lead to cell death. Importantly, as cancer cells exhibit increased levels of endogenous DNA damage due to an excessive replication stress, inhibiting the checkpoint kinases alone could act as a directed anti-cancer therapy. Here, we review the current status of inhibitors targeted towards the checkpoint effectors and discuss mechanisms of their actions in killing of cancer cells.

## 1. Introduction

**Figure 1:**
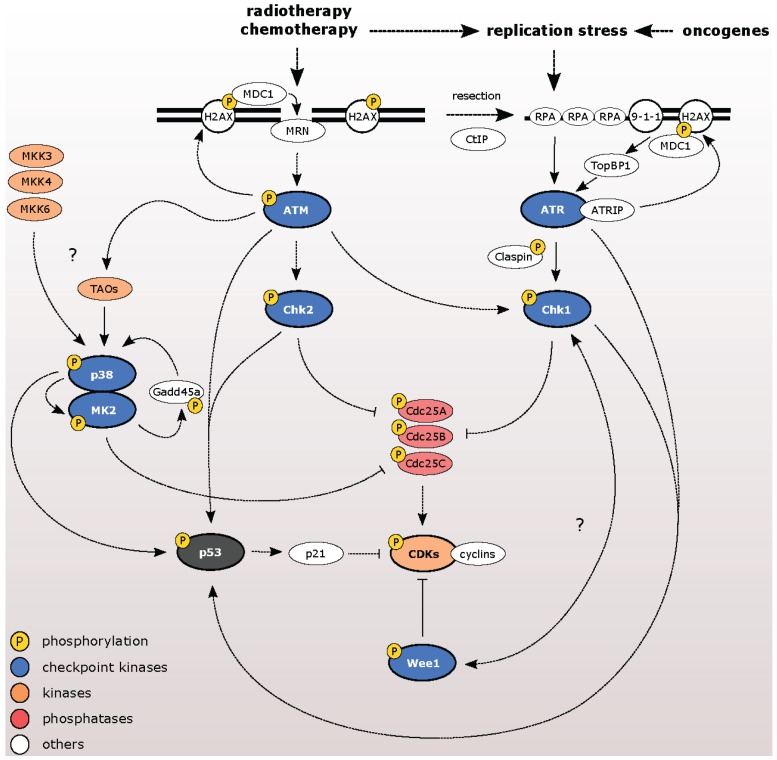
DNA damage response activates cell cycle checkpoint. Cell cycle progression is controlled by CDKs that are inactivated by Wee1 kinase and activated by Cdc25A/B/C phosphatases. Induction of DSBs activates ATM/Chk2 pathway, while exposed ssDNA initiates the ATR/Chk1 pathway. Both pathways inactivate Cdc25A/B/C leading to a temporal cell cycle arrest. Activation of p53/p21 pathway is crucial for the G1 checkpoint. The activation of the p38MAPK/MK2 pathway contributes to the checkpoint activation and maintenance.

Cells cope with genotoxic stress by triggering a signaling network termed as DNA damage response (DDR) that coordinates the cell cycle progression and DNA repair (Figure 1). Double-strand DNA breaks (DSBs) activate ataxia-telangiectasia mutated kinase (ATM). During S and G2 phases of the cell cycle, DNA flanking the DSB undergoes resection in order to promote error-free repair through homologous recombination (HR). Exposed stretches of single-stranded DNA are coated by RPA that recruits and activates a complex of ataxia-telangiectasia-mutated and Rad 3-related kinase (ATR) with its cofactor ATRIP (reviewed in [1]). In addition, ATR is activated by single stranded DNA (ssDNA) during replication and, to a higher extent, by stalled replication forks during replication stress. ATM and ATR activate checkpoint kinases Chk2 and Chk1, respectively. Cyclin-dependent kinases (CDKs) control progression through the cell cycle and are regulated by inhibitory phosphorylations at residues Thr-14 and Tyr-15 by Wee1 and Myt1 kinases and their dephosphorylation by Cdc25 phosphatases. Phosphorylation and inhibition of Cdc25A/B/C phosphatases by Chk1 and Chk2 leads to efficient inhibition of CDKs and triggers a temporal checkpoint arrest (reviewed in [2]). Aside from canonical checkpoint kinases, the p38MAPK-MK2 pathway also contributes to checkpoint activation. Particularly, MK2 shares substrate homology with Chk1 and Chk2 and can inactivate Cdc25 phosphatases in a similar manner [3,4,5]. The establishment of the checkpoint relies mostly on posttranslational modifications of the effector proteins and, therefore, can occur rapidly after DNA damage. With slower kinetics, DDR activates the tumor suppressor p53 pathway that involves changes in the expression of a large number of target genes. Following DNA damage, p53 is phosphorylated at multiple residues by ATM/ATR, Chk1/Chk2, and p38 and this leads to its stabilization. In turn, p53 triggers transcription of a potent CDK inhibitor p21 that is crucial for the G1 checkpoint. In addition, p21-dependent inhibition of CDK also contributes to the maintenance of the G2/M checkpoint by transcriptional repression of cyclin B and Plk1 [6,7].

The checkpoint arrest gives cells time to repair the damaged DNA. After completion of DNA repair, cells recover from the checkpoint and continue in the progression through the cell cycle. If damage exceeds the repair capacity, cells remain permanently arrested in senescence or they are eliminated by programmed cell death. Importantly, the induction of genotoxic stress by ionizing radiation or by chemotherapy represents a major non-surgical mode of cancer treatment. In addition, several genetic defects present in a subset of tumors can potentially be exploited for design of personalized treatment strategies [8]. One of such promising examples is represented by tumors with deficient homologous recombination due to the mutations in *BRCA1* or *BRCA2*. These tumors show a high sensitivity to poly(ADP-ribose)polymerase inhibitors that block alternative DNA repair pathways and may help to kill BRCA1-deficient cancer cells (recently reviewed in [9,10,11]). Here, we will discuss other possibilities how pharmacological regulation of the DDR pathway and cell cycle checkpoints can increase the sensitivity of cancer cells to therapy.

## 2. Checkpoint Inhibition as a Directed Anti-Cancer Therapy

### 2.1. Sensitizing Cancer Cells to DNA Damaging Agents with Checkpoint Inhibitors

**Figure 2:**
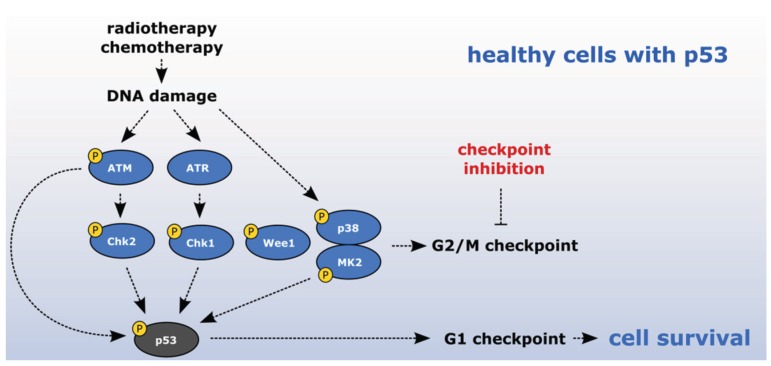

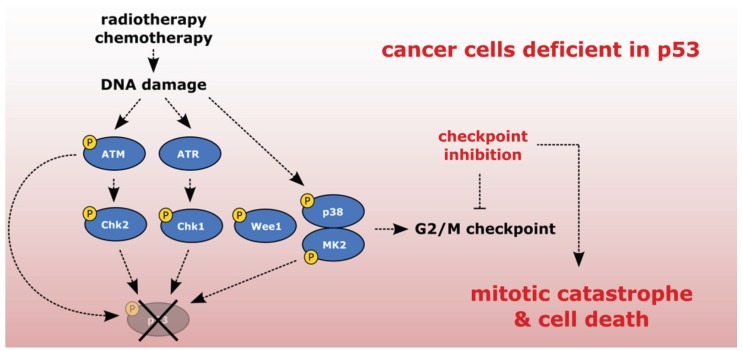
Sensitizing cancer cells to DNA-damaging agents with checkpoint inhibitors. Cancer cells deficient in p53 lack G1 checkpoint and depend on checkpoint kinases to establish G2/M checkpoint. Inhibition of checkpoint kinases in combination with DNA damaging therapy leads to the G2/M checkpoint abrogation, mitotic catastrophe and cell death. Notably, healthy cells are protected by p53-dependent response.

Cell cycle checkpoints protect the genome integrity and at organismal level oncogene-induced senescence (OIS) acts as a barrier preventing tumor development [12,13]. During tumorigenesis, cells acquire mutations that allow them to partially bypass the checkpoints and avoid establishing the OIS. Cancer cells harboring a deficient p53 pathway lack efficient G1 checkpoint and maintaining the G2/M checkpoint fully depends on checkpoint kinases. Checkpoint abrogation promotes mitotic entry despite the presence of DNA damage, which results in mitotic catastrophe and cell death [14]. Therefore, the pharmacological inhibition of checkpoint kinases in combination with the DNA damaging chemotherapy or radiotherapy was proposed to represent a promising cancer treatment strategy [15]. Tumor cells that are already deficient in p53, lose the remaining protective effect of checkpoint kinases and, thus, become hypersensitive to chemotherapeutics, whereas healthy cells are still protected by the p53-dependent response (Figure 2). Such combined therapy should, hence, result in mild side-effects towards healthy cells while efficiently eradicating the cancer cells. Indeed several studies demonstrated that sensitization by checkpoint inhibitors allowed to reduce doses of DNA damaging chemotherapeutics and thus decrease the normal tissue toxicity [16,17,18,19]. The pharmacological approaches to inhibit particular kinases are discussed in detail below.

### 2.2. Exploiting the Addiction of Hyper-Replicating Cancer Cells to ATR-Chk1-Wee1 Signaling

Replication stress is now recognized as one of the cancer hallmarks [20]. While a low level of replication stress is inherent to normal DNA replication, a high level of replication stress represents a pathological condition connected to cancer development. Activation of oncogenes such as Ras, Myc, and Cyclin E leads to the aberrant increase of CDK activity, increased DNA replication origin firing and to replication stress [12,13]. Replication stress is usually represented by stalling of replication forks followed by their collapse and conversion into DSBs. An excessive replication stress is caused by the global increase of origin licensing or firing, and is consequently further pronounced at the level of individual replicating forks by the relative deficiency of the nucleotides [20]. In addition, it has recently been shown that the nuclear pool of RPA represents a limiting factor in hyper-replicating cells [21]. The excess of ssDNA generated during hyper-replication can exhaust the pool of available RPA, which can no longer protect ssDNA regions at ongoing replication forks. This, consequently, results in a genome-wide DNA breakage, termed a replicative catastrophe [21].

The exact mechanism of how the exposed ssDNA at the site of stalled forks is converted into chromosomal breaks is not fully understood. It has been suggested that DSBs can arise during the attempts to resolve the stalled forks by homologous recombination (HR) [22]. In this scenario, the stalled forks are firstly cleaved by a complex of MUS81-EME1 endonucleases and then repaired by classical HR [23]. It is possible that in case of an excessive number of stalled forks, this activity ultimately results in unrepaired DSBs. Supporting this view, depletion of MUS81 and EME1 has been shown to increase resistance to replication stress [24].

**Figure 3:**
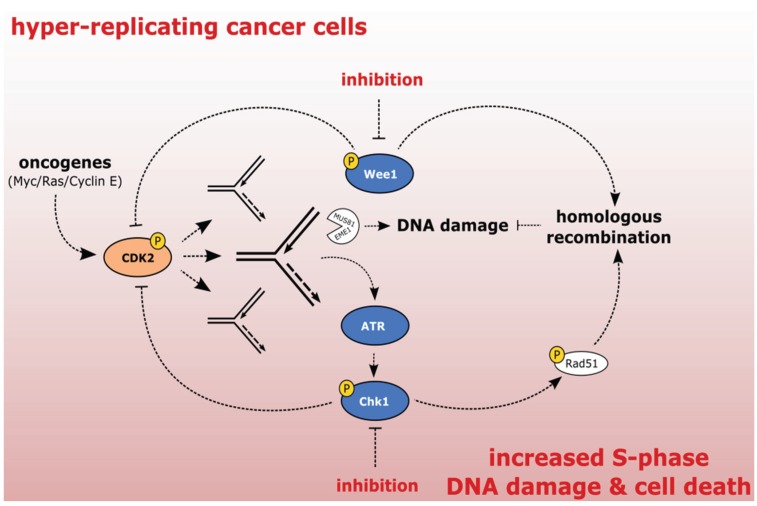
Exploiting the addiction of cancer cells to ATR-Chk1-Wee1 signaling. The activation of oncogenes results in increased CDK activity, hyper-replication, and replication stress. Stalled forks are converted to DSBs. ATR/Chk1/Wee1 kinases oppose CDK2 activation and protect cells from the excessive replication stress. Chk1 and Wee1 protect cells from DNA damage by promoting homologous recombination (HR). Inhibition of ATR/Chk1/Wee1 kinases in cancer cells leads to excessive DNA damage and cell death.

Under normal conditions, the level of replication stress is under tight control of ATR/Chk1 and Wee1 kinases that counterbalance the activation of CDK2 and ensure a proper DNA replication rate during the S-phase progression [22]. CDK2 activity is down-regulated by phosphorylation of Tyr-15 by Wee1. In addition, Chk1-dependent degradation of Cdc25A further inhibits the CDK2 activity and suppresses the origin firing. Loss of ATR, Chk1, or Wee1 leads to an excessive exposure of ssDNA, followed by a vast DNA breakage and cell death. Consistent with essential functions of ATR, Ckh1, and Wee1 in replication, mice knockouts show early embryonic lethality [25,26,27]. However, a partial inhibition of these kinases can be tolerated by normal cells, whereas it can efficiently eradicate cancer cells [28] (Figure 3). Melanoma and *MYC-*driven lymphoma cells exhibit high levels of replication stress and show excellent sensitivity to Chk1 inhibitors *in vitro,* suggesting that these cancer types might be suitable candidates for testing the efficacy of single agent treatment *in vivo* [29,30]. The cytotoxic effect can be explained by the addiction of hyper-replicating cancer cells to the ATR/Chk1/Wee1 signaling that protects them from replicative catastrophe. Consistent with this hypothesis, increased expression of ATR/Chk1/Wee1 kinases was reported in various cancer cell lines [31,32].

### 2.3. Exploiting the Deficient HR Pathway for Increased Sensitivity of Cancer Cells

Homologous recombination is an error-free DNA repair pathway that can occur only during S and G2 phases when the replicated sister chromatid is available and can serve as a template. To allow the proper coordination of HR in context of the cell cycle, the signaling pathway that controls HR is also strictly regulated by CDKs and checkpoint kinases. Resection of DSBs is possible only after phosphorylation of CtIP by CDK2 [33,34]. In addition, Chk1 has been shown to be directly involved in HR through a direct phosphorylation of Rad51 at Thr-309, which is necessary for Rad51 recruitment to the sites of DNA damage [35]. Similarly, Wee1 promotes HR by down-regulating the CDK1-dependent inhibitory phosphorylation of Brca2 at Ser-3291 [36]. Significant numbers of human tumors are deficient in homologous recombination. The most common examples are represented by the inactivating mutations in *BRCA1/2* and *RAD51* in breast and ovary cancer [37,38].

Numerous recent studies have demonstrated that tumor cells with deficient HR are highly sensitive to PARP inhibition (reviewed in [10]). Unfortunately, subsequent clinical trials revealed that treatment with PARP inhibitors commonly leads to the development of resistance and to the relapse of tumor growth. In genetically-unstable tumors this is mainly enabled by the accumulation by additional mutations (such as in *TP53BP1*) that eventually allow a partial rescue of the HR and thus limit a therapeutic response to PARP inhibition [9,11].

Deficient HR limits the rate of replication fork restart and, thus, also leads to induction of the replication stress. To cope with the increased replication stress, *BRCA1/2*-mutated ovarian cancers commonly amplify *ATR* and *CHK1* genes [31]. Importantly, depletion or inhibition of Rad51 dramatically increased the sensitivity of ovarian cancer cells to ATR and Chk1 inhibition, suggesting that HR deficiency and inhibition of ATR/Chk1 pathway can be synthetically lethal [31].

### 2.4. Exploiting the Deficient G2 Checkpoint in Targeting Cancer Cells

As discussed above, activation of the G1 checkpoint is commonly impaired in cancer cells due to the loss of p53. On the other hand, some cancer types are deficient in the G2 checkpoint which can also affect their sensitivity to pharmacological intervention. A substantial fraction of melanoma cells fails to arrest in the G2 checkpoint and shows increased sensitivity to histone deacetylase and PI3K kinase inhibitors *in vitro* [39,40], recently reviewed in [41]. The ability of these drugs to efficiently suppress melanoma growth *in vivo,* as well as the potential use of these inhibitors in targeting other cancer types, still needs to be experimentally tested.

## 3. Pharmacological Inhibitors of Checkpoint Kinases

### 3.1. ATM Kinase

DNA double strand breaks activate the ATM kinase. The site of DSB is recognized by the MRN complex (composed of Mre11, Rad50, and NBS1 subunits) that recruits ATM to the damage site [42,43]. ATM phosphorylates histone H2AX at Ser-139 in the vicinity of the break, which is subsequently bound by MDC1 that further amplifies the signal by recruiting more MRN molecules [44,45]. Chromatin in the vicinity of the lesion is extensively modified further and attracts repair factors such BRCA1 and 53BP1 (reviewed in [1]). The active ATM phosphorylates Chk2 at Thr-68 and, thus, activates a diffusible checkpoint effector kinase Chk2 [46].

Mutations that impair function of ATM kinase cause ataxia-telangiectasia syndrome (A-T) that involves cerebellar degeneration, immunodeficiency, hypersensitivity to radiation, and increased incidence of cancer. The observed hypersensitivity of A-T patients to radiation points out the ATM as a promising target for radiosensitization and chemosensitization in cancer therapy. The first drugs inhibiting ATM described to radiosensitize cells were caffeine and wortmannin [47,48]. Nevertheless both represent largely unspecific drugs that inhibit all members of the PI3K kinase family and show high toxicity *in vivo*. Several specific inhibitors of ATM have been developed—KU-55933 (IC_50_ = 13 nM) [49], CP466722 [50], KU-60019 [51] and KU-559403 [52]. While the first three drugs exhibit high specificity and potency *in vitro*, all demonstrate poor bio-availability *in viv*; albeit not a perspective for the clinical administration, use of these drugs had proven the principle of radiosensitization by ATM inhibition. Exposure of transformed cells to KU-55933 caused significant sensitization to both ionizing radiation and chemotherapeutic agents such as etoposide, doxorubicin, and camptothecin [49,53]. Further structure optimization resulted in the development of more effective second generation ATM inhibitor KU-60019 (IC_50_ = 6.3 nM) that was proven to radiosensitize glioblastoma cells *in vitro* and when injected directly into a tumor it also markedly radiosensitized glioma xenografts in mice [51,53,54,55,56]. Notably, glioma xenografts derived from the isogenic cell line with inactivated p53 were much more sensitive to the treatment with KU-60019 and radiation than their p53 wild-type counterparts [56]. KU-559403 is the first specific inhibitor of ATM that shows good solubility and good tissue distribution necessary for *in vivo* use. KU-59403, alone, had no impact on tumor growth but significantly enhanced cytotoxicity of camptothecin, etoposide, and doxorubicin *in vitro,* and similarly enhanced the antitumor activity of camptothecin and irinotecan in colon cancer xenografts in mice [52]. In contrast to other ATM inhibitors, the sensitizing effect of KU-59403 on the growth of colon cancer xenografts was not dependent on the p53 status [52]. It is currently unclear whether this reflects a particular cancer type (colon cancer *vs.* glioblastoma), type of p53 mutation (deletion *vs.* point mutation) or different administration scheme. It will also be interesting to see how these potent ATM inhibitors will perform in clinical trials.

### 3.2. ATR Kinase

ATR signaling is triggered by various DNA lesions that expose ssDNA, including resected ends of DSBs, ssDNA gaps generated during DNA repair, and stalled or collapsed replication forks [57]. The DSBs are processed during S and G2 phases the by endonucleases Mre11 and CtIP into single-stranded 3' overhangs [58,59]. The resection of DSBs end promotes both DNA repair by homologous recombination and activation of ATR pathway. CtIP-mediated resection was described to be particularly important for sustained ATR-Chk1 checkpoint signaling and for maintenance of the intra-S and G2-phase checkpoints [60]. Exposed ssDNA is immediately coated by RPA [61] which is, in turn, recognized by a stable complex of ATR-ATRIP [62]. This leads to ATR accumulation at the site of DNA lesion. ATR catalytic activity is further potentiated by its interaction with TopBP1 that is recruited to the site of lesion either by 9-1-1 complex, scaffold protein RHINO, or by its direct interaction with MDC1 [63,64,65,66,67]. ATR-ATRIP complex interacts with, and phosphorylates, adaptor protein Claspin that directs ATR activity towards Chk1 [68,69]. ATR phosphorylates C-terminal regulatory domain of Chk1 on several SQ/TQ sites, including Ser-317 and Ser-345, that are thought to be indispensable for Chk1 activation [70].

Since ATR is an essential gene, it has been anticipated that pharmacological inhibition of ATR may not be well tolerated *in vivo*. However, several recent studies demonstrate that cancer cells may be considerably more sensitive to the partial ATR inhibition compared to healthy cells [71,72,73]. The increased sensitivity to hypomorphic ATR reduction was observed in sarcomas expressing active forms of H-RasG12V, acute myeloid leukemia driven by *MLL-ENL*, and in *c-MYC*-driven lymphoma [71,72,73]. Interestingly, increased sensitivity of cancer cells to the partial reduction of ATR occurred irrespectively of the p53 status, suggesting that ATR inhibition could be efficient also in oncogene-transformed tumors with compromised p53 pathway [73]. In addition, osteosarcoma and glioblastoma cancer cells that proliferate due to the activation of alternative lengthening of telomeres (ALT) pathway have recently been reported to be hypersensitive to ATR inhibitors [74]. Finally, several genetically-determined defects in DNA repair (such as loss of *XRCC1* or translesion polymerase *REV3L*) are synthetically lethal in combination with the treatment with ATR inhibitors and cisplatin [75,76].

VE-821 was the first specific and potent ATR inhibitor that abrogated G2/M checkpoint and reduced survival of various cancer cell lines after radiotherapy or treatment with chemotherapeutics including cisplatin, etoposide, gemcitabine, neocarzinostatin, and camptothecin [77,78,79,80,81,82,83]. The sensitizing effect of VE-821 was further pronounced in p53- or ATM-deficient backgrounds [78,82]. A close analogue of VE-821, named VE-822 (or alternatively CX970), with even increased potency against ATR was shown to radiosensitize and chemosensitize pancreatic cancer cells *in vitro* and pancreatic tumor xenografts *in vivo* [84]. VE-822 also potentiated the effect of cisplatin in primary human lung tumor cells *in vitro* and in patient-derived lung tumor xenografts [85]. Currently, the VE-822 is tested in combination with gemcitabine, cisplatin, and etoposide in a phase I clinical trial.

Another class of potent and specific ATR inhibitors is represented by AZ20 and its analogue AZD6738 (IC_50_ = 1 nM) with improved solubility, pharmacodynamics, and bioavailability [86,87]. The AZD6738 has recently entered the phase I clinical trials in monotheraphy, in combination with carboplatin, olaparib, and with radiotherapy.

### 3.3. Chk1 and Chk2 Kinases

Chk1 kinase is activated upon its phosphorylation by ATR on Ser-317 and Ser-345 [88]. Subsequently, autophosphorylation of Chk1 at Ser-296 creates a docking site for 14-3-3g that mediates interaction between Chk1 and its substrate Cdc25A [89]. Cdc25A phosphorylated at Ser-76 is subsequently ubiquitinated by SCF/βTrCP ubiquitin ligase complex and degraded by proteasome [90,91,92]. In addition, Chk1 phosphorylates and, thus, inhibits actions of Cdc25B and Cdc25C phosphatases. Cdc25B is phosphorylated at Ser-323 and bound by 14-3-3 that blocks its catalytic activity [93]. Cdc25C is phosphorylated at Ser-216, recognized by 14-3-3 and sequestered in the cytoplasm, which prevents its actions towards nuclear CDKs [94]. Activation of Chk2 is triggered by ATM-dependent phosphorylation at Thr-68, followed by several autophosphorylation events [46,95]. Albeit structurally distinct, Chk2 shares the substrate homology with Chk1 and inhibits Cdc25A/B/C phosphatases in a similar way. Nevertheless, Chk1 and Chk2 are not functionally interchangeable. Whereas Chk1 is viewed as the main executory checkpoint kinase, Chk2 may act as a signal booster and seems to be, at least under certain conditions, redundant [96].

The first and most studied inhibitor of Chk1 is UCN-1 (also known as staurosporine). Notably, UCN-1 represents largely non-specific inhibitor that inhibits to significant extent several cellular kinases (IC_50_ for Chk1 11 nM, Chk2 1040 nM, CDK1 31 nM, CDK2 30 nM, PKC 7 nM, and MK2 95 nM) [97,98]. The fact that UCN-01 also targets CDK kinases could, in principle, hinder its effect on checkpoint abrogation. Nonetheless, it has been shown that UCN-01 overrides the G2/M checkpoint upon treatment with DNA damaging agents such as cisplatin or topoisomerase inhibitor SN-38 [99,100]. UCN-01 was tested in a number of clinical trials as a single agent or in combination with genotoxic insults (https://clinicaltrials.gov/). The conducted trials showed that UCN-01 has undesirably-long half-life and decreased bioavailability due to its binding human plasma protein α1-acid glycoprotein [101] and harmful side-effects, reflecting its poor specificity [102]. Currently the use of UCN-1 as anti-cancer drug seems unlikely [103].

In addition, several ATP-competitive inhibitors that show similar efficiency to Chk1 and Chk2 are available. Among these, AZD7762 [18], XL844 [104], and PF00477736 [17] significantly increased the sensitivity of cancer cells to gemcitabine *in vitro* and were tested in phase I clinical trials. However, clinical testing of AZD7762 was stopped due to reported cardiac toxicity [105] and evaluation of PF00477736 and XL844 was also prematurely terminated.

Second generation of Chk1 inhibitors shows improved selectivity towards Chk1. The first selective Chk1 inhibitor that entered clinical trials was LY2603618 (IC_50_ = 7 nM) [106]. In preclinical studies LY2603618 was described to abrogate the G2/M checkpoint upon treatment with doxorubicin and gemcitabine. Consistent with previous studies on Chk1 inhibition, human lung cancer cells with mutant p53 showed increased sensitivity to the combined treatment with LY2603618 and gemcitabine in xenograft model [107]. In this trial, a partial response was observed in two out of 14 non-small cell lung cancer patients treated with combination of LY2603618, folate antimetabolite pemetrexed and cisplatin [108].

MK-8776 (SCH 900776) is a novel selective Chk1 inhibitor (IC_50_ = 3 nM) that sensitized cancer cells to gemcitabine and hydroxyurea [19,28]. Results from a phase I trial suggest that MK-8776 is well-tolerated in monotherapy and also in combination with gemcitabine. This phase I clinical trial also reported the first promising evidence of clinical efficacy of the MK-8776 treatment in patients with advanced solid tumors [109]. MK-8776 is currently being tested in a phase II clinical trial in patients with refractory acute leukemia.

### 3.4. Wee1 Kinase

Expression level of Wee1 kinase increases during S and G2 phases of the cell cycle [110]. Upon mitotic entry, Wee1 is degraded by SCF/βTrCP in a Plk1- and Cdk1-dependent manner [111]. After DNA damage, Xenopus Wee1 is phosphorylated by Chk1 at Ser-549 that increases its inhibitory kinase activity towards CDKs [112]. Nevertheless, such regulation of Wee1 by Chk1 has not been described in human cells yet. Wee1 phosphorylates CDKs at Tyr-15 in a vicinity of its ATP-binding pocket and thus inhibits its activity [110,113]. Wee1 is required for sustained ATR/Chk1 activity upon replication stress [114]. Thus, inhibition of Wee1 after the gemcitabine treatment increases Cdk1 activity, which impairs DNA resection by CtIP and weakens the activation of ATR. In addition, Wee1 inhibition mediates inactivation of Chk1 through the Plk1-dependent decrease of Claspin levels [114].

Several Wee1 inhibitors have been developed. PD0166285 represents a nonselective tyrosine kinase inhibitor, which targets Wee1 but also Chk1, Myt1, c-Src, PDGFR-β, fibroblast growth factor receptor-1, and epidermal growth factor receptor tyrosine kinases [115,116]. PD0166285 inhibits CDK1 phosphorylation at Tyr-15 and Thr-14 and abrogates G2/M checkpoint upon irradiation *in vitro* [116]. PD407824 is a dual inhibitor of Chk1 and Wee1 and was shown to sensitize cancer cells to cisplatin and gemcitabine [117,118].

MK-1775 (AZD1775) is currently the most advanced specific inhibitor of Wee1 [119,120]. MK-1775 selectively sensitizes p53-deficient cancer cells to gemcitabine, carboplatin, 5-fluorouracil and cisplatin [119,121]. MK-1775 abrogates the radiation-induced G2/M checkpoint in p53-deficient cells but not in p53 wild-type cells [122]. Moreover, MK-1775 synergizes with radiotherapy and gentamicin treatment to regress p53-deficient xenografts, as opposed to the xenografts with wild-type p53 [122,123]. Taken together, preclinical studies have shown that MK-1775 significantly and selectively sensitizes p53-defective cancer cells to DNA-damaging agents both *in vitro* and *in vivo*. MK-1775 is currently evaluated in several clinical trials, either as a single agent or in combination with DNA-damaging agents (https://clinicaltrials.gov). Paired tumor biopsies (before and after MK-1775 treatment) showed that MK-1775 decreased CDK1-Tyr15 phosphorylation and increased H2AX phosphorylation [124]. This observation is in line with the preclinical studies, in which inhibition of Wee1 induced replication stress and DNA damage. More importantly, partial clinical response was reported in two out of 25 patients with *BRCA-1/2* deficient solid tumors treated with MK-1775 [124].

Although the mutual regulation between Wee1 and Chk1 in human cells remains unclear, there is increasing evidence that combined inhibition of Wee1 and Chk1 synergizes in cytotoxic effect. Inhibition of both Chk1 and Wee1 was shown to cause aberrant replication, impaired G2/M checkpoint, premature entry to mitosis before completion of replication and, ultimately, abnormal mitosis and cell death [125]. In addition, combined inhibition of Wee1 and Chk1 efficiently inhibited tumor growth in various xenograft models including ovarian cancer, neuroblastoma, mantle cell lymphoma, and melanoma [125,126,127,128].

### 3.5. p38/MK2 Kinases

The p38 mitogen-activated protein kinase/MAPKAP kinase-2 (MK2) pathway responds to a wide range of stress stimuli, including osmotic stress, oxidative stress, heat shock, inflammatory cytokines, and DNA damage. Molecular mechanism that couples DNA damage with p38/MK2 pathway has not been fully elucidated but, at least in some contexts, ATM/ATR can contribute to p38 activation [129,130]. The active p38 phosphorylates MK2 at Thr-222 and Thr-334 leading to its activation [131]. MK2 shares a substrate homology with Chk1 and contributes to the establishment of checkpoint by phosphorylation of Cdc25 [98]. In addition, p38/MK2 pathway was implicated in the G2/M checkpoint maintenance [132]. Upon DNA damage, p38/MK2 complex translocates from the nucleus to the cytoplasm where MK2 phosphorylates hnRNPA0 leading to the stabilization of Gadd45a mRNA. In a positive feedback loop, Gadd45a potentiates p38-mediated MK2 activation, which allows sustained activation of the G2/M checkpoint [132].

Importantly, combined depletion of MK2 and doxorubicin treatment abrogated G2/M checkpoint and caused mitotic catastrophe in p53^−/−^ MEFs [98]. Moreover, loss of MK2-sensitized p53-deficient non-small-cell lung cancer tumors to cisplatin in mice suggesting that synthetic lethality between p53 and MK2 can be exploited for sensitization of tumors to DNA-damaging chemotherapy *in vivo* [133]. Screening for synthetic lethality has recently revealed that combined inhibition of MK2 and Chk1 synergistically induced mitotic catastrophe and cell death in *KRAS-* and *BRAF*-driven cancer cells [134]. The same synergistic interaction was observed in xenografts, autochthonous KRAS-driven lung adenocarcinomas in mice, and tumor cells isolated from cancer patients. As *KRAS* expression leads to increased replication stress, the effect of combined MK2 and Chk1 inhibition likely take advantage of the addiction of hyper-replicating cancer cells to checkpoint signaling.

## 4. p53 Pathway Modulators

Tumor suppressor *TP53* is the most commonly mutated gene in human solid tumors and inactivating mutations in *TP53* are commonly associated with bad prognosis in cancer patients receiving conventional therapies (reviewed in [135]). At the same time however, loss of p53 can be exploited for designing novel treatment strategies relying on increased sensitivity of p53-deficient cancer cells to checkpoint inhibition. On the other hand, many other cancers types (such as neuroblastoma, hematologic malignancies and melanoma) contain mutated *TP53* much less frequently. These p53-proficient cancers can be sensitized to chemotherapy by further boosting up the p53 response, which ultimately promotes p53-dependent cell cycle arrest or apoptosis. This approach is well supported in mouse models, where restoration of p53 function leads to tumor regression [136].

Chemosensitization of p53 wild type cancer cells by Mdm2 antagonists has been proven in preclinical studies and is currently tested in clinical trials (reviewed in [137]). Nutlin-3 (*cis*-imidazoline, RG7112) inhibits the interaction between p53 and its negative regulator Mdm2 (IC_50_ = 0.09 μM) resulting in stabilization of p53 and high expression of its transcriptional targets [138]. Nutlin-3 strongly suppresses growth of p53 wild-type cancer cells and induces apoptosis [138]. RG7388 is a novel derivate of *cis*-imidazoline with improved bioavailability that efficiently induced apoptosis in p53 wild type neuroblastoma cell lines *in vitro* and blocked tumor growth in xenograft models [139,140,141].

An alternative target for chemosensitization of p53 wild-type tumors is protein phosphatase magnesium-dependent 1 delta (*PPM1D*, referred to as Wip1) that dephosphorylates p53 on Ser-15 and acts as its negative regulator. Loss of *PPM1D* strongly delayed development of oncogene-induced tumors in mice [142,143]. Down-regulation of Wip1 by RNA interference increased apoptosis in various p53^+/+^ cancer cell lines [144,145]). Until recently, development of specific Wip1 inhibitors remained a major challenge. GSK2830371 is a novel allosteric inhibitor of Wip1 that efficiently inhibits growth of p53^+/+^ hematopoietic tumor cell lines *in vitro* and in xenograft models [146]. In addition, GSK2830371 suppressed growth of neuroblastoma cells and potentiated thy cytotoxic effect of doxorubicin and carboplatin [147]. Wip1 is commonly overexpressed or stabilized by truncating mutations in several cancer types, including breast and ovary cancer, glioblastoma, and medulloblastoma [148,149]). Further studies are now needed to identify cancer types that will respond well to the Wip1 inhibitors and also to determine the efficacy of Wip1 inhibitors *in vivo*.

## 5. Targeting Cyclin-Dependent Kinases in Cancer Therapy

Cell proliferation is tightly regulated by cyclin-dependent kinases suggesting their potential use as pharmacological targets in cancer therapy [150,151]. Whereas CDK1/cyclin B play indispensable roles in cell cycle progression, development, and tissue homeostasis, other CDKs and cyclins are not essential for proliferation in healthy tissues [151]. In contrast, there is now emerging evidence that survival of several cancer types depends critically on specific interphase cyclins and CDKs. This has been first demonstrated for cyclin D1 and CDK4/6 that are required for development of *RAS*- and *HER2*-driven mammary tumors and *KRAS-*induced lung tumors [152,153,154]. Similarly, cyclin D3 and CDK6 were essential for development of T-cell leukemia and Burkitt lymphoma [155,156]. In addition, activity of CDK4/6 is frequently up-regulated by deletion of *CDKN2A* (p16INK4a) or increased expression of cyclin D1 in melanoma [157,158,159].

The first generation of pan-CDK inhibitors, such as Flavopiridol (Alvocidib) or second generation multi-CDK inhibitors such as P276-00, were shown to induce cell cycle arrest and apoptosis in cancer cells *in vitro* and cause regression of xenografts in mice [160,161,162]. Nonetheless, the therapeutic use of non-selective CDK inhibitors is limited due to the severe toxicity and lack of specificity. In striking contrast, Palbociclib (PD0332991), a highly specific inhibitor CDK4 (IC_50_ = 11 nM) and CDK6 (IC_50_ = 16 nM), is well tolerated and has shown to efficiently suppress growth of estrogen receptor-positive breast cancer [163,164], non-small cell lung carcinoma [154], and various hematologic malignancies including T cell acute lymphoblastic leukemia [164,165], Burkitt lymphoma [156], and mantle cell lymphoma [166]. Promising results from the phase II studies in estrogen receptor-positive breast cancer have been recently reported for two other CDK4/6 selective inhibitors, LEE011 (ribociclib) and LY2835219 (abemaciclib) [167]. Whereas loss of CDK4/6 activity induced senescence in melanoma and breast cancer [164,168], it induced apoptosis in leukemia [164], suggesting that Notch-driven malignancies might be particularly sensitive to CDK4/6 inhibition. In addition, CDK4/6 kinases have been proposed as promising pharmacological targets in *BRAF* inhibitor-resistant melanoma [169].

## 6. Conclusions

**Figure 4:**
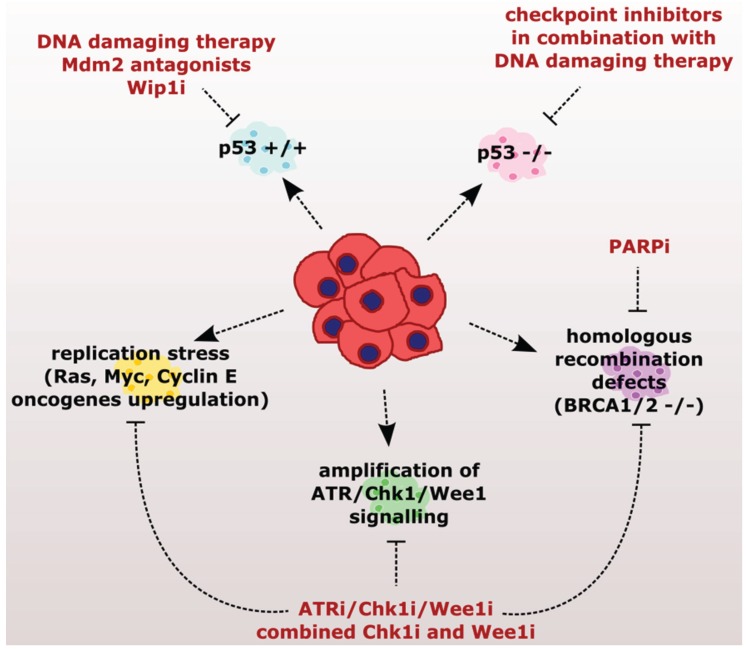
Targeting the weak spots in individual tumors. Individual tumors exhibit various genetic backgrounds and thus have different weak spots. Single targeted therapy can never be efficient in all possible tumor variants. Therefore there is a great need to introduce diagnostic approaches that would, firstly, identify the weak spots of the particular tumor and then choose the effective targeted therapy.

Extensive amounts of preclinical data highlight the potential use of small-molecule inhibitors of the checkpoint kinases for targeted cancer therapy (Figure 4 and Table 1). Encouraged by the efficiency to eradicate cancer cells *in vitro* and in mouse models, several inhibitors are currently tested in clinical trials. The first approach combines inhibition of the checkpoint kinases with conventional DNA damaging therapies. In this case cancer cells lacking the G1 checkpoint lose the remaining protective effect of the G2/M checkpoint and die by mitotic catastrophe. The efficiency of this approach depends on the status of the p53 pathway and, thus, loss of p53 represents a main predictive marker for the response to the combined treatment with DNA-damaging reagents and checkpoint inhibitors. The second strategy relies on addiction of cancer cells transformed by active oncogenes (such as Ras, Myc or Cyclin E) to ATR, Chk1, and Wee1 kinases that allow them to cope with a high level of replication stress. These cancer cells are more sensitive to the partial inhibition of ATR/Chk1/Wee1 kinases compared to the healthy counterparts. In this case, increased sensitivity does not depend on p53 and, therefore, could be widely used without determining the status of *TP53.* On the other hand, inhibitors of ATR/Chk1/Wee1 will likely be efficient only in tumors with the high level of replication stress. Therefore, reliable biomarkers of replication stress are needed for prediction of the treatment response. Suitable candidates include histone H2AX phosphorylated at Ser-139 (general marker of genotoxic stress), Chk1 phosphorylated at Ser-345 (active form of Chk1), and Cdc25 phosphorylated at Ser-216 (marker of active checkpoint) that all score positive in cells undergoing replication stress. The positivity of these markers correlated well with the response of the *c-Myc*-driven large B-cell lymphoma to Chk1 inhibition [170]. The third strategy exploits genetically-determined defects in DNA repair pathways (mostly homologous recombination) that render cancer cells more sensitive to PARP or ATR/Chk1/Wee1 inhibitors. Deficient HR can be deduced from identified inactivating mutations of already known genes (such as *BRCA1* or *RAD51*) in tumor biopsies; however, the major challenge is represented by prediction of the drug resistance development. Finally, tumors that retain intact p53 could be sensitized by increasing p53 levels through antagonizing Mdm2 or by inhibition of Wip1 phosphatase.

Underlying mechanisms of the DNA damage response and checkpoint control are now understood at a molecular level, as well as their synergistic effects in targeting cancer cells. Recently-applied synthetic lethality screening approaches are likely to identify novel interactions between various cell cycle checkpoint components that can be exploited for targeted cancer therapy. New generations of pharmacological inhibitors to the checkpoint kinases show dramatically improved substrate specificity and bioavailability, which will hopefully reduce possible off-target effects and allow administration at efficient doses, respectively. In addition to the checkpoint regulation, our increasing understanding of the specific roles of CDKs in cancer cells survival further broadens the possibilities of cancer therapy. Successful implementation of the novel cancer treatment strategies into clinics will depend on the development of reliable biomarkers suitable for classification of the tumors and allowing selection of patients responsive to the checkpoint inhibitors.

**Table 1:** Selected inhibitors of checkpoint kinases.

Inhibitor	Targeted kinase(s)	Reference
KU-55933	ATM (IC_50_ = 13 nM)	[49,53]
	DNA-PK (IC_50_ = 2.5 μM)	
	PI3K (IC_50_ = 16.6 μM)	
	mTOR (IC_50_ = 9.3 μM)	
KU-60019	ATM (IC_50_ = 6.3 nM)	[51,53,54,55]
KU-59403	ATM (IC_50_ = 3 nM)	[52]
	DNA-PK (IC_50_ = 9.1 μM)	
	PI3K (IC_50_ = 10 μM)	
	mTOR (IC_50_ = 14 μM)	
VE-821	ATR (IC_50_ = 13 nM)	[77,78,80,81,82,83]
	ATM (IC_50_ = 16 μM)	
	DNA-PK (IC_50_ = 2.2 μM)	
	PI3K (IC_50_ = 3.9 μM)	
VE-822 (CX970)	ATR (IC_50_ = 19 nM)	[84,85]
AZ20	ATR (IC_50_ = 5 nM)	[86]
AZD6738	ATR (IC_50_ = 1 nM)	[87]
UCN-1 (staurosporine)	Chk1 (IC_50_ = 11 nM)	[97,98,99,100,101,102]
	MK2 (IC_50_ = 95 nM)	
	Chk2 (IC_50_ = 1 μM)	
	PKC (IC_50_ = 7 nM)	
	CDK1 (IC_50_ = 31 nM)	
	CDK2 (IC_50_ = 30 nM)	
LY2603618	Chk1 (IC_50_ = 7 nM)	[106,107]
XL844 (EXEL-9844)	Chk1 (IC_50_ = 2.2 nM)	[104]
	Chk2 (IC_50_ = 200 nM)	
AZD7762	Chk1 (IC_50_ = 5 nM)	[18]
	Chk2 (IC_50_ = 5 nM)	
PF00477736	Chk1 (K_i_ = 0.49 nM)	[17]
	Chk2 (K_i_= 47 nM)	
MK-8776 (SCH-900776)	Chk1 (IC_50_ = 3 nM)	[19,28,109]
	Chk2 (IC_50_ = 1.5 μM)	
PD0166285	Wee1 (IC_50_ = 24 nM)	[115,116]
	Myt1 (IC_50_ = 72 nM)	
PD407824	Wee1 (IC_50_ = 97 nM)	[117,118]
	Chk1 (IC_50_ = 47 nM)	
MK-1775 (AZD1775)	Wee1 (IC_50_ = 5.2 nM)	[119,120,121,122,123]
